# Retraction: The Exponentiated Exponential Burr XII distribution: Theory and application to lifetime and simulated data

**DOI:** 10.1371/journal.pone.0253706

**Published:** 2021-06-21

**Authors:** The PLOS ONE Editors

Following publication of this article [1], concerns were raised about overlap in content between this article and a previously published work by the first author [2]. The corresponding author requested retraction of the article.

Having confirmed the overlap, and in accordance with the request of the corresponding author, the *PLOS ONE* Editors retract this article, as we consider that this constitutes redundant publication.

This article [1] reports material from [2], published 2021 IOS Press, which is not offered under a CC-BY license. The retracted *PLOS ONE* article is therefore not offered under the Creative Commons Attribution License. At the time of retraction, the article [1] was republished to update its copyright statement.

MI agreed with retraction and apologizes for the issues with the published article. MB either could not be reached or did not respond directly.

Update: The retracted *PLOS ONE* article was removed from the *PLOS ONE* website on May 11, 2022 and the removed contents are no longer offered under the Creative Commons Attribution License. Readers should refer to [2] for the copyright notice.
